# Fruit ripening-associated leucylaminopeptidase with cysteinylglycine dipeptidase activity from durian suggests its involvement in glutathione recycling

**DOI:** 10.1186/s12870-021-02845-6

**Published:** 2021-02-01

**Authors:** Pawinee Panpetch, Supaart Sirikantaramas

**Affiliations:** 1 Molecular Crop Research Unit, Department of Biochemistry, Faculty of Science, Chulalongkorn University, 254 Phayathai Road, Bangkok, 10330 Thailand; 2 Omics Sciences and Bioinformatics Centre, Chulalongkorn University, 254 Phayathai Road, Bangkok, 10330 Thailand

**Keywords:** Cys-Gly, Durian, Fruit ripening, LAP, Leucylaminopeptidase, Sulphur compound

## Abstract

**Background:**

Durian (*Durio zibethinus* L.) is a highly popular fruit in Thailand and several other Southeast Asian countries. It is abundant in essential nutrients and sulphur-containing compounds such as glutathione (GSH) and γ-glutamylcysteine (γ-EC). Cysteinylglycine (Cys-Gly) is produced by GSH catabolism and occurs in durian fruit pulp. Cysteine (Cys) is a precursor of sulphur-containing volatiles generated during fruit ripening. The aforementioned substances contribute to the strong odour and flavour of the ripe fruit. However, the genes encoding plant Cys-Gly dipeptidases are unknown. The aim of this study was to measure leucylaminopeptidase (LAP) activity in durian fruit pulp.

**Results:**

We identified DzLAP1 and DzLAP2, which the former was highly expressed in the fruit pulp. *DzLAP1* was expressed at various ripening stages and in response to ethephon/1-MCP treatment. Hence, *DzLAP1* is active at the early stages of fruit ripening. DzLAP1 is a metalloenzyme ~ 63 kDa in size. It is activated by Mg^2+^ or Mn^2+^ and, like other LAPs, its optimal alkaline pH is 9.5. Kinetic studies revealed that DzLAP1 has K_m_ = 1.62 mM for its preferred substrate Cys-Gly. DzLAP1-GFP was localised to the cytosol and targeted the plastids. *In planta* Cys-Gly hydrolysis was confirmed for *Nicotiana benthamiana* leaves co-infiltrated with Cys-Gly and expressing *DzLAP1*.

**Conclusions:**

DzLAP1 has Cys-Gly dipeptidase activity in the γ-glutamyl cycle. The present study revealed that the LAPs account for the high sulphur-containing compound levels identified in fully ripened durian fruit pulp.

**Supplementary Information:**

The online version contains supplementary material available at 10.1186/s12870-021-02845-6.

## Key message

DzLAP1 characterisation in durian (*Durio zibethinus* L.) fruit pulp indicated that it participates in cysteinylglycine degradation and glutathione recycling during ripening.

## Background

Durian (*Durio zibethinus* L.) is a highly flavourful fruit grown in Thailand and other Southeast Asian countries. Fresh durian production and export are highly profitable. Several studies showed that durian is rich in protein, carbohydrate, fat [1], and bioactive phenolic and anti-proliferative compounds [2–4]. Durian fruit has unique texture, flavour, and odour. During ripening, the sulphur-containing volatile-associated gene methionine γ-lyase (*MGL*) and the ethylene-related gene aminocyclopropane-1-carboxylic acid synthase (*ACS*) are upregulated. Correspondingly, their respective metabolites (sulphur volatiles and ethylene) accumulate. Hence, there is a correlation between ethylene biosynthesis and sulphur volatile production during ripening [5]. After the publication of the draft durian genome in 2017 [5], our group identified several transcription factors associated with ripening process. For example, DzDof2.2 is involved in the regulation of auxin biosynthesis [6] and DzARF2A could bind to the promoters of several ethylene biosynthetic genes and, thus, controls durian fruit ripening [7].

Several sulphur-containing volatiles are responsible for the pungent odour of ripe durian fruit. Glutathione (GSH), gamma-glutamylcysteine (γ-EC), *S*-adenosylmethionine (SAM), cysteine (Cys), and cysteinylglycine (Cys-Gly) were detected in the ripe fruit pulp of the Thai cultivars Chanee and Monthong [8]. The former has a stronger odour and faster postharvest ripening than the latter. Moreover, ripe Chanee fruit has higher Cys and Cys-Gly levels than ripe Monthong fruit [8].

Cys-Gly is an important intermediate in the γ-glutamyl cycle of sulphur metabolism. It participates in redox homeostasis and recycles amino acids in living cells. In mammals, Cys-Gly is the product of GSH degradation via sequential γ-glutamyl transpeptidase (GGT), γ-glutamyl cyclotransferase (GGCT), and 5-oxoprolinase (OPase) reactions [9]. Nevertheless, only the concerted actions of GGCT and OPase or a GGT-independent pathway governed Cys-Gly production in *Arabidopsis* [10]. Cys-Gly is hydrolysed to Cys and Gly by a dipeptidase. Cys-Gly is a pro-oxidant and may contribute to the intracellular redox environment. Excess Cys-Gly was toxic to yeast cells [11, 12]. Therefore, enzymatic Cys-Gly regulation may be vital. Moreover, the release of Cys is important as this amino acid can be converted to methionine used in sulphur volatile production during fruit ripening [5].

Leucylaminopeptidases (LAPs, EC. 3.4.11.1) are members of the M17 enzyme family. They might process intracellular proteins but their precise metabolic functions are still unknown. They preferentially hydrolyse Cys-Gly in *Bos taurus* (cow) [13], *Treponema denticola* [14], and *Arabidopsis* [15]. They were originally named LAPs as early reports suggested that they react with *N*-terminal leucines [16]. The cytosolic M20 metallopeptidase Dug1p has Cys-Gly peptidase activity. Its homologues occur in mammals and yeast but not plants [17]. LAPs have six identical monomers comprising two conserved Zn-binding sites per subunit [18]. They all participate in amino acid turnover but their other biological functions are complex and species-specific. *Escherichia coli* LAP, also known as XerB, PepA, or CarP, is an aminopeptidase-independent DNA-binding protein [19, 20]. It mediates site-specific plasmid and phage recombination [21, 22] and activates transcriptional *carAB* operons [20]. Multiple functions have been reported for mammalian LAPs. Interferon-γ (IFN-γ) induction promoted high LAP accumulation. LAP may participate in antigen presentation in humans [23, 24]. Animal LAPs have been implicated in oxidative lens aging [25]. Plant LAP-A is a defence protein that plays important roles in floral development in Solanaceae [26–29].

Plant LAPs are either acidic LAP-A or neutral LAP-N depending on their pI. LAP-A and LAP-N have distinct biochemical properties and respond differently to developmental and environmental cues. LAP-A occurs only in the Solanaceae, is induced by biotic and abiotic stress [30–32], and accumulates in reproductive organs [33, 34]. In contrast, LAP-N is constitutively produced in all plants [32, 34]. *LAP-A*-silenced tomatoes were relatively more susceptible to *Manduca sexta* (tomato hornworm) invasion than their wild type counterparts [35]. LAP-A and LAP-N are molecular chaperones protecting proteins from heat damage [36]. Here, we identified two LAP isoforms in durian fruit pulp. Of these, the LAP-N DzLAP was highly expressed in the pulp. We biochemically characterised DzLAP1 expressed as a His-tagged protein in *E. coli.* Our objective was to clarify its cooperative function in sulphur-volatile production via Cys liberation from Cys-Gly cleavage. We discovered that DzLAP1 was localised to both the cytoplasm and chloroplast. We examined its physiological roles in cytosolic GSH and plastidial peptide catabolism. To the best of our knowledge, the present work is the first to report the involvement of LAPs and associate DzLAP1 with durian fruit ripening.

## Results

### *LAP* identification, protein sequence alignment, and phylogenetic analysis

**Fig. 1:**
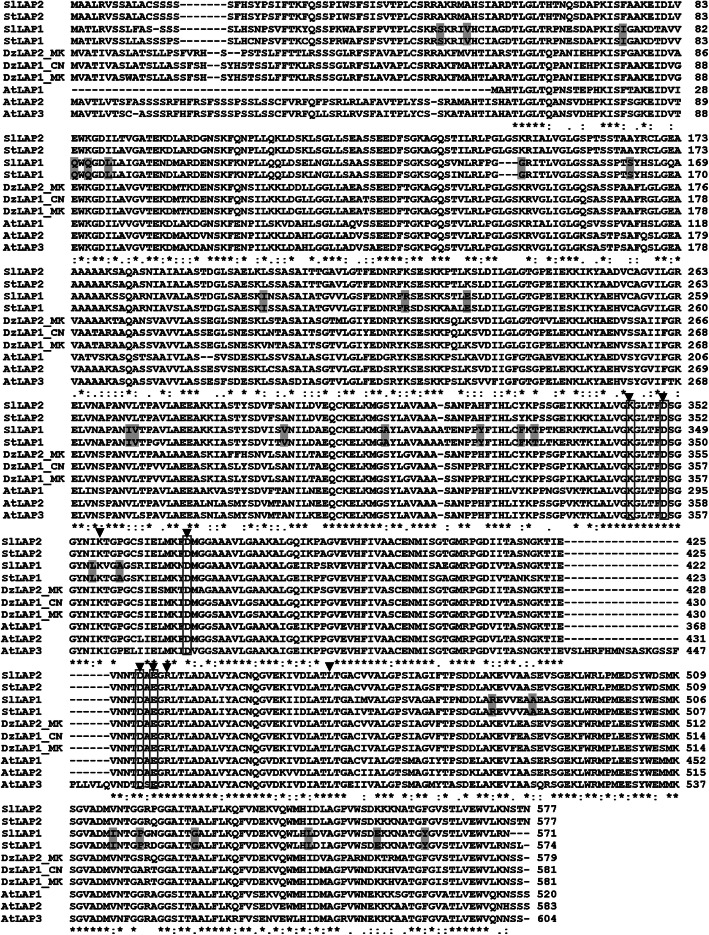
Amino acid sequence alignment of various plant LAPs. Deduced amino acid sequence of Chanee DzLAP1 (DzLAP1_CN) aligned with Musang King DzLAP1_MK and DzLAP2_MK (*Durio zibethinus*), *Arabidopsis* (*Arabidopsis thaliana*) AtLAP1–AtLAP3; potato (*Solanum tuberosum*) StLAP1 and StLAP2; and tomato (*Solanum lycopersicum*) SlLAP1 and SlLAP2. Eight substrate binding/catalytic residues are marked by arrowheads. Five of the eight residues are conserved metal ion-coordinating residues (boxes). Twenty-eight LAP-A signature residues are highlighted. Asterisks, colons, and dots indicate strictly, highly, and moderately conserved amino acid residues, respectively

According to the open-source Musang King durian cultivar genome database, *DzLAP1_MK* and *DzLAP2_MK* were identified. Only one Chanee *DzLAP* isoform was present in the in-house RNA-seq data obtained from ripe fruit pulp tissues (data not shown). Full-length Chanee DzLAP was aligned with the LAPs of Musang King and tomato (*Solanum lycopersicum*; SlLAP1 and SlLAP2), potato (*Solanum tuberosum*; StLAP1 and StLAP2), and *Arabidopsis* (*Arabidopsis thaliana*; AtLAP1 to AtLAP3). The putative Chanee *DzLAP* was annotated as *DzLAP1* (accession no. MN879753) and encoded DzLAP1. It was represented as DzLAP1_CN in multiple alignment and phylogenetic neighbour-joining (NJ). DzLAP1_CN clustered with Musang King DzLAP1_MK. DzLAP1 shared 99.3 and 89.7% identity with DzLAP1_MK and DzLAP2_MK, respectively. The essentially high % identity between DzLAP1 and DzLAP1_MK is possibly resulted from the SNP present among different cultivars. There were eight highly conserved residues (K350, D355, K362, D375, D435, E437, R439, and L463 of DzLAP1) involved in substrate binding or catalytic function (Fig. 1, arrowheads). Five conserved residues (K350, D355, D375, D435, and E437) interacted with metal ions [37, 38] which are vital enzyme cofactors. These conserved residues comprise a subset of the catalytic residues (Fig. 1, boxes). All protein sequences including DzLAP1 but neither SlLAP1 nor StLAP1 harboured substitutions of all 28 LAP-A signature residues (Fig. 1, highlights). Fully conserved residues were detected in the *C*-terminal region containing all essential active residues participating in catalysis (Fig. 1, asterisks). In contrast, the *N*-terminal region harboured highly variable amino acid sequences [34]. AtLAP1 lacked the signal peptide sequence but the *N*-terminal regions of SlLAPs and StLAPs were slightly shorter than the other sequences. Only ~ 64–77% identity was established when comparing two isoforms of both SlLAP and StLAP with two and three isoforms of DzLAP_MK and AtLAP, respectively.

**Fig. 2:**
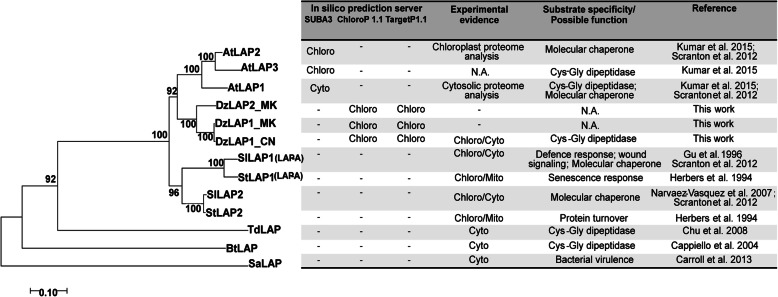
Phylogenetic analysis and comparison of various LAPs. A neighbour-joining (NJ) tree was constructed with MEGA v. 7 using 1000 bootstrap replicates. *Arabidopsis thaliana*: AtLAP1 (NP_179997), AtLAP2 (NP_194821), and AtLAP3 (NP_001328632); *Solanum lycopersicum*: SlLAP1 (NP_001233862.2) and SlLAP2 (NP_001233884.2); *Solanum tuberosum*: StLAP1 (XP_006350102.1) and StLAP2 (XP_015165363.1); *Durio zibethinus:* Musang King DzLAP1_MK (NW_019167860.1) and DzLAP2_MK (NW_019168159) and Chanee DzLAP1_CN (MN879753); *Treponema denticola*: TdLAP (WP_010698434.1); *Bos taurus*: BtLAP (NP_776523.2); *Staphylococcus aureus*: SaLAP (ORO33369.1). The table on the right represents the locations of LAPs analysed either by in silico prediction or empirical evidence and based on their putative roles

A phylogenetic analysis revealed that all plant LAPs clustered together and were separate from those of bacteria (Fig. 2). However, Cys-Gly peptidase activity was detected in certain plant and bacterial LAPs. Tomato SlLAPs and potato StLAPs were separate from those of *Arabidopsis* and durian and participated in defence responses and protein catabolism (Fig. 2). In contrast, they had little activity towards Cys-Gly [30]. Various LAPs resided in different cellular compartments and may have had divergent putative functions (Fig. 2).

### Gene expression analysis by qRT-PCR

**Fig. 3:**
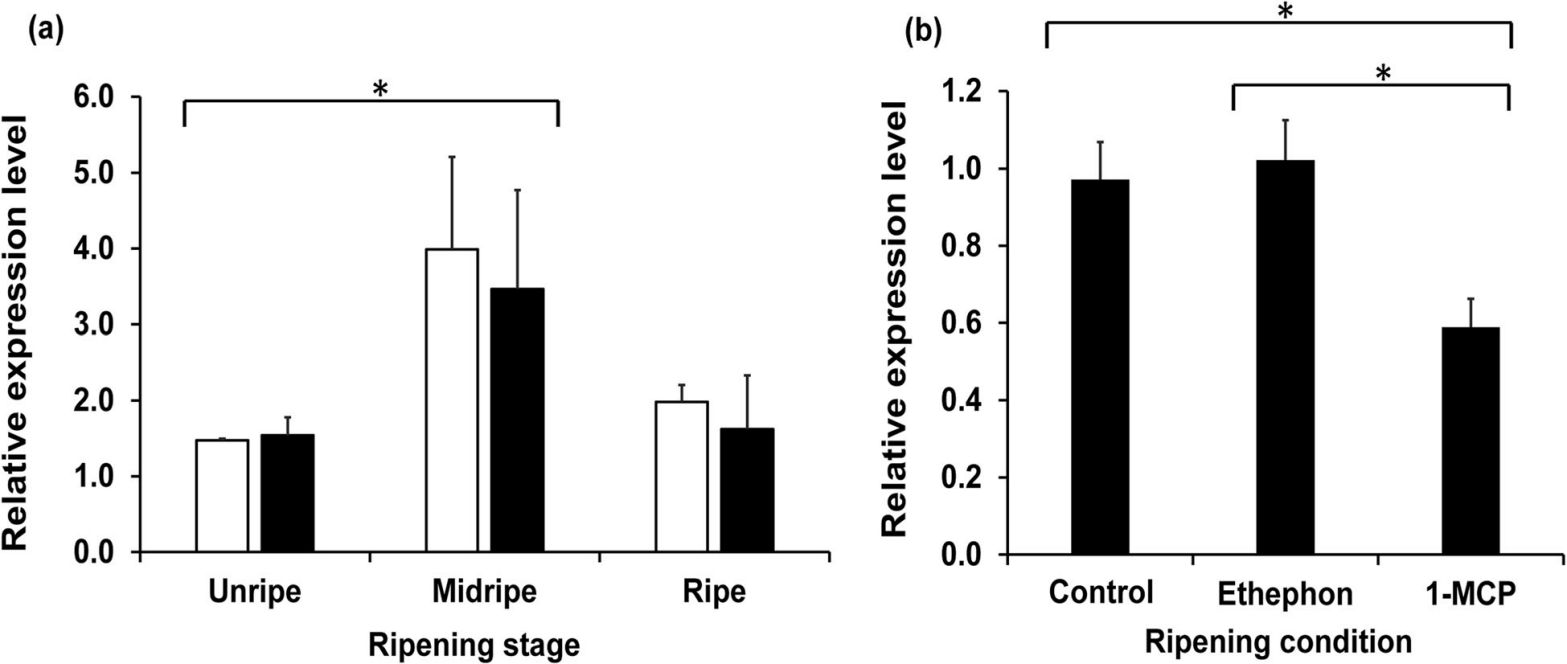
qRT-PCR analysis of *DzLAP1* at various stages of Chanee and Monthong durian cultivar fruit ripening (**a**) and under different Monthong durian fruit cultivar ripening conditions (**b**). *DzLAP1* expression in durian fruit pulp at unripe, midripe, and ripe stages in Chanee (white bars) and Monthong (black bars) cultivars (**a**) and under natural ripening (control), ethephon treatment, and 1-MCP-treatment (**b**). Elongation factor 1 alpha (*EF-1α*) was the reference gene. Bars: means ± standard deviation (SD) of three independent biological replicates. Asterisks indicate significant differences based on Tukey’s HSD multiple-range test (*p* < 0.05)

In silico analysis disclosed that *DzLAP_MK* expression substantially varied among tissue types. *DzLAP1_MK* expression was highest in durian pulp whereas *DzLAP2_MK* was expressed in other tissues (Supplementary Fig. S1). Hence, we focused on *DzLAP1_MK* as it was nearly identical to Chanee *DzLAP1*. The qRT-PCR revealed differential *DzLAP1* expression at the unripe, midripe, and ripe stages of Chanee and Monthong fruit pulp*. DzLAP1* was significantly upregulated from the unripe to midripe stages but downregulated by the ripe stage (Fig. 3a). *DzLAP1* expression in the Chanee cultivar (white bars) somewhat resembled that in the Monthong cultivar (black bars) (Fig. 3a). The phytohormones ethephon and 1-MCP were applied to validate the association between *DzLAP1* and fruit ripening. *DzLAP1* was significantly downregulated in the fruit pulp treated with 1-MCP compared to the fruit pulp undergoing natural ripening (control) or treated with the ripening agent ethephon (Fig. 3b). However, ethephon treatment had relatively little influence on *DzLAP1* expression.

### *In planta* Cys-Gly dipeptidase activity analysis

**Fig. 4:**
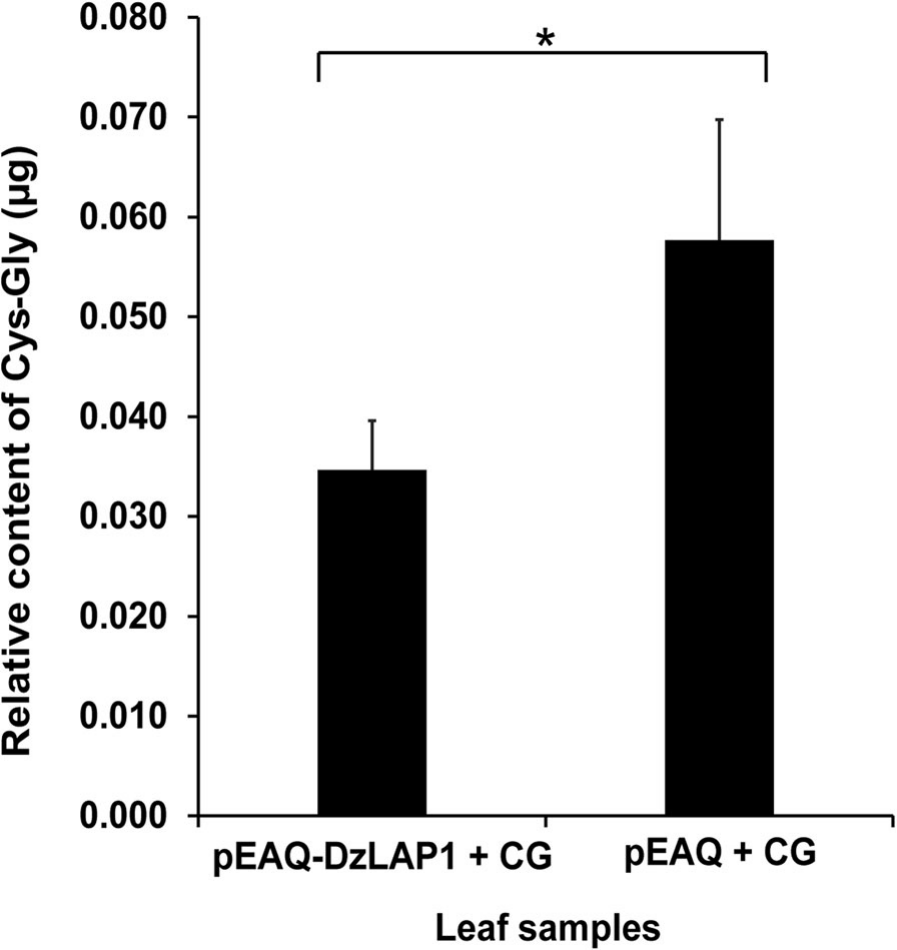
*In planta* Cys-Gly dipeptidase activity assay in *DzLAP1*-overexpressing *N. benthamiana* leaves. Leaves co-infiltrated with *A. tumefaciens* GV3101 harbouring pEAQ-*DzLAP1* or pEAQ empty vector + 15 mM Cys-Gly were extracted with 0.1 M HCl (ratio of 1 mg/50 μL). The internal standard was 10 mM *N*-acetylcysteine. The relative Cys-Gly content is shown. Samples were three independent biological replicates obtained from separate leaves. Student’s *t*-test (*p* < 0.05) identified significant differences (asterisks) between *DzLAP1*-overexpressing and control leaves

Cys-Gly dipeptidase activity was assessed for crude fruit pulp extracts at the unripe and midripe stages (Supplementary Fig. S3). Cys-Gly dipeptidase activity was significantly higher in the midripe (white bars) than the unripe (black bars) sample (*p* < 0.05). This finding was consistent with that obtained from the gene expression analysis (Fig. 3a). Cys-Gly dipeptidase activity was also confirmed for *Nicotiana benthamiana* leaves co-infiltrated with 15 mM Cys-Gly and harbouring either pEAQ-*DzLAP1* or pEAQ (control). The leaves were collected and their Cys-Gly levels were quantified by HPLC. The Cys-Gly was significantly higher in the control than the *DzLAP1*-overexpressing leaves (*p* < 0.05) (Fig. 4).

### In vitro rDzLAP1 production and biochemical characterisation

**Fig. 5:**
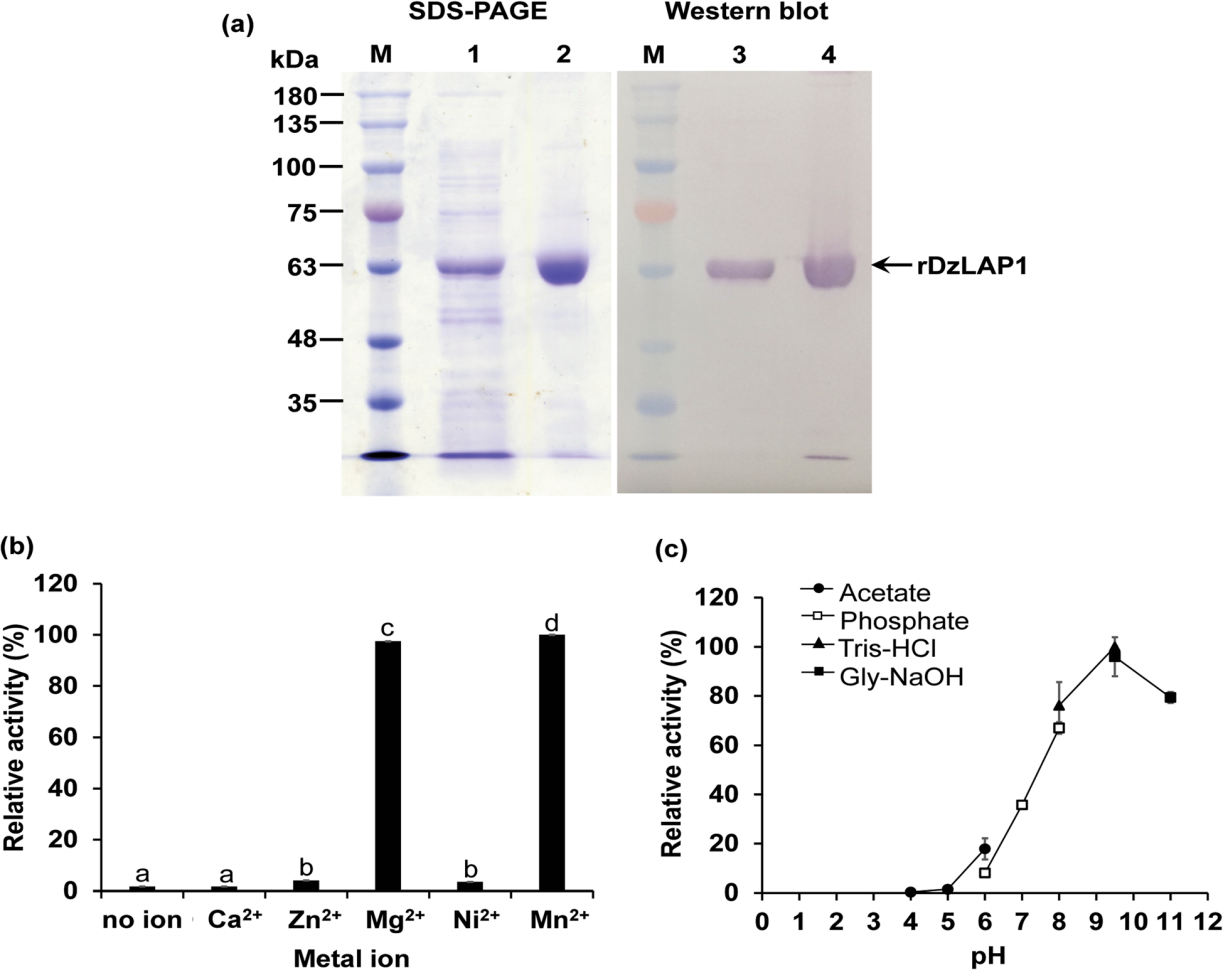
SDS-PAGE and western blot (**a**) metal ion dependency (**b**) and optimal pH (**c**) of recombinant DzLAP1. Lane M: standard protein ladder. Lanes 1 and 3: crude rDzLAP1. Lanes 2 and 4: purified rDzLAP1. Two micrograms crude and purified proteins were loaded onto SDS gel (**a**). Three and one-half micrograms rDzLAP1 was incubated with 7.5 mM Cys-Gly in 25 mM K_3_PO_4_ buffer (pH 7.2) in the presence of 0 mM and 1 mM Ca^2+^, Zn^2+^, Mg^2+^, Ni^2+^, and Mn^2+^ at 37 °C for 15 min. The total reaction volume was 50 μL. Enzyme activity was measured spectrophotometrically by a modified acidic ninhydrin method [39] at 560 nm (A_560_) (**b**). The optimum pH for rDzLAP1 was established by incubating 3.5 μg enzyme with 7.5 mM Cys-Gly and 1 mM Mg^2+^ at various pH (acetate buffer, pH 4–6; phosphate buffer, pH 6–8; Tris-HCl buffer, pH 8–9.5; glycine-NaOH buffer, pH 9.5–11) at 37 °C for 15 min (**c**). Enzyme activity was calculated relative to a maximum of 100%

Purified soluble rDzLAP1 produced by *E. coli* appeared as a single-band protein on SDS gel. Its molecular weight was ~ 63 kDa and was confirmed by western blot (Fig. 5a). Relative to the BSA standard, the purified DzLAP1 concentration was ~ 1.16 mg mL^− 1^.

The metalloenzyme activity of DzLAP1 was tested by incubating the enzyme with Cys-Gly substrate in the presence of Ca^2+^, Zn^2+^, Mg^2+^, Ni^2+^, and Mn^2+^. DzLAP1 activity was measured by a modified acidic ninhydrin method detecting released cysteines are detected. The highest DzLAP1 activity was obtained when the reaction systems contained Mg^2+^ and Mn^2+^. Conversely, Ca^2+^, Zn^2+^, and Ni^2+^ had minimal effect as DzLAP1 activity in their presence did not significantly differ from that measured for the metal ion-free control (Fig. 5b). Mg^2+^ was selected as the DzLAP1 cofactor in the enzyme kinetics assay. Optimal DzLAP1 pH was established by incubating the enzyme with Cys-Gly at pH 4.0–11.0. DzLAP1 had maximum activity against Cys-Gly at pH 9.5 (Fig. 5c) and ~ 80% of the enzyme activity occurred in a pH range of 8.0–11.0. At pH < 7.0, DzLAP1 activity was < 50% and at pH 4.0–5.0, the enzyme was inactive.

To identify enzyme substrate specificity, 3.5 μg purified rDzLAP1 was incubated with various substrate concentrations in the presence of 1 mM MgCl_2_ + 25 mM K_3_PO_4_ buffer (pH 8.0) at 37 °C for a specific length of time. There was no DzLAP1 activity in the presence of GSH or γ-Glu-Cys (Table 1). DzLAP1 had positive activity against various α-linked dipeptides. Therefore, it was proposed that this enzyme is a Cys-Gly dipeptidase as it had maximum catalytic efficiency in the presence of Cys-Gly. The *k*_*cat*_/K_m_ for Cys-Gly was ~ 118× and ~ 6× higher than those for Met-Gly and Leu-Gly, respectively (Table 1). However, a previous report indicated that LAP preferred *N*-terminal Leu peptides and had high affinity for Leu-Gly (K_m_ = 0.35 mM) [30].

**Table 1:** DzLAP1 kinetics on different substrates

Substrate	***k***_***cat***_ (min^**− 1**^)	K_**m**_ (mM)	***k***_***cat***_/K_**m**_ (min^**− 1**^ mM^**− 1**^)
GSH	N.D.	N.D.	N.D.
γ-Glu-Cys	N.D.	N.D.	N.D.
Cys-Gly	74.8 ± 8.1	1.6 ± 0.3	46.2
Met-Gly	2.1 ± 0.4	5.2 ± 1.3	0.4
Leu-Gly	2.9 ± 0.4	0.4 ± 0.1	8.2

### Subcellular DzLAP1 localisation in *N. benthamiana*

**Fig. 6:**
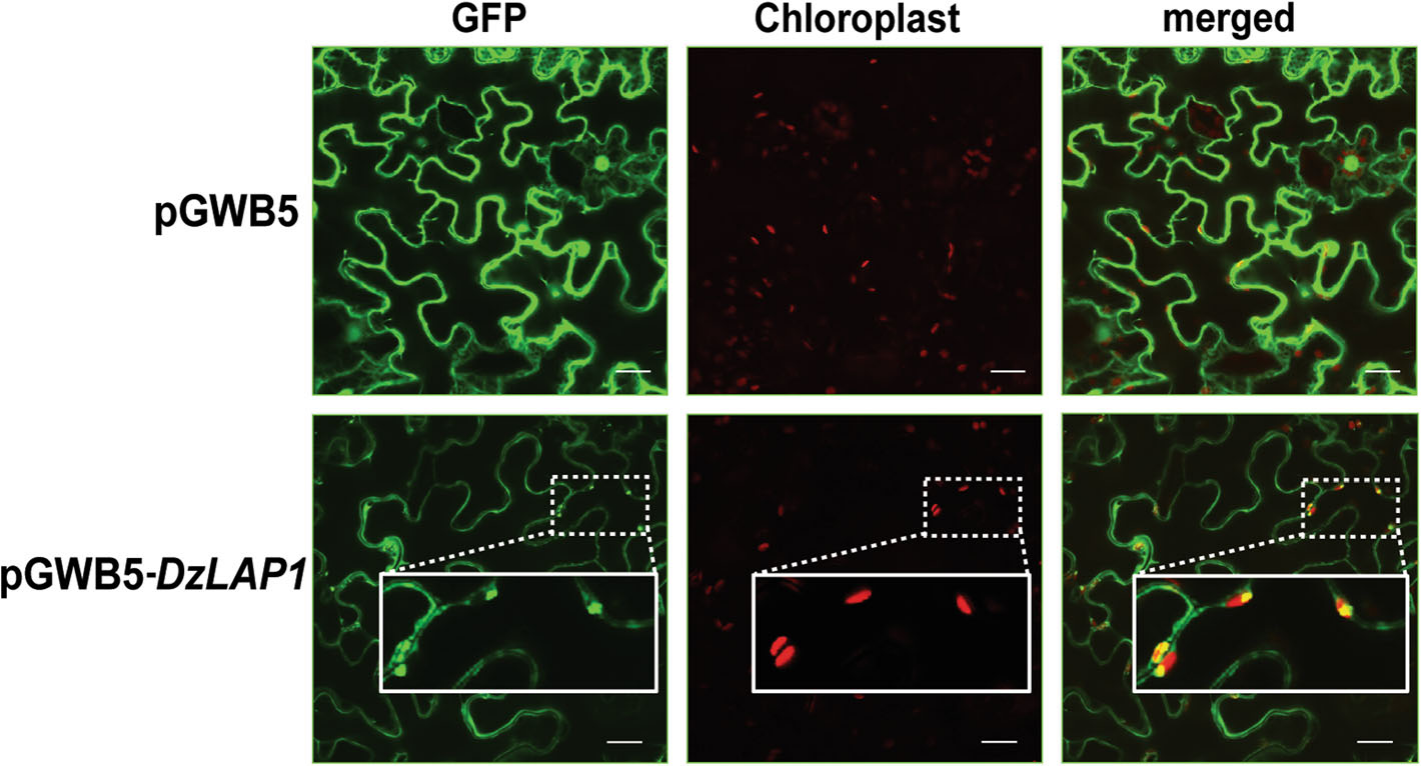
Subcellular GFP-tagged DzLAP1 localisation in *Nicotiana benthamiana* leaves. Confocal microscopic images of *N. benthamiana* leaf epidermal cells infiltrated with pGWB5 (control; upper panel) and pGWB5-*DzLAP1* (lower panel). GFP fluorescence (GFP), chloroplast autofluorescence (chloroplast), and merged images are shown. Plastidial DzLAP1-GFP localisation is shown as enlargements in the insets. Scale bars: 20 μm

An in silico analysis predicted that DzLAP1 is a chloroplast-localised protein. The pGWB5-*DzLAP1* and the silencing suppressor *p19* were co-expressed in *Agrobacterium tumefaciens* GV3101 infiltrated in 4-wk *N. benthamiana* leaves. The *in planta* assay showed that GFP-tagged DzLAP1 was a soluble protein probably localised to the cytosol (Fig. 6). Fluorescence signals were detected in the chloroplasts (Fig. 6, insets). Hence, durian DzLAP1 may be localised either to cytosol or the chloroplasts.

## Discussion

Durian fruit pulp accumulates large quantities of GSH [8] which is vital for plant cell homoeostasis [40] and stores Cys via incorporation with Glu and Gly in the γ-glutamyl cycle [41]. Cys is recycled from the hydrolysis of Cys-Gly which is a by-product of GSH breakdown. This mechanism may generate abundant ethylene precursors and sulphur-volatile compounds via Met synthesis. Both pathways promote durian fruit ripening and the malodour associated with it. Cys-Gly was detected in durian fruit pulp [8]. Hence, the γ-glutamyl cycle must be activated in durian fruit ripening. In the present study, we aimed to identify and characterise the DzLAPs involved in the aforementioned biochemical processes. There has been little published information on the involvement of Cys-Gly dipeptidase in the γ-glutamyl cycle [15, 17].

LAPs are highly conserved metallopeptidases in animals, plants, and microorganisms [42]. Several *LAP*s were identified in *Arabidopsis* [15] and durian (this work). *Arabidopsis* has three *LAP*s encoding AtLAP1–AtLAP3. *DzLAP1_MK* and *DzLAP2_MK* were detected in the durian cultivar Musang King genome [5]. We focused on *DzLAP1_MK* as it was highly expressed in durian fruit pulp (Supplementary Fig. S1) and GSH and γ-EC accumulated there [8]. DzLAP1_MK showed 99.3% identity with DzLAP1 in the durian cultivar Chanee. DzLAP1 was the only isoform detected in our in-house RNA-seq data. A primary protein sequence analysis (Fig. 1) confirmed that DzLAP1 is LAP-N as it contains the conserved substrate binding, catalytic, and metal ion-binding residues associated with the enzyme mechanism.

Postharvest *DzLAP1* expression analyses showed upregulation at the midripe stage. Thus, DzLAP1 may hydrolyse Cys-Gly to Cys and Gly and cause the strong malodour associated with durian fruit pulp ripening. Relative *DzLAP1* expression was similar for Chanee and Monthong at all ripening stages (Fig. 3a). However, the Cys-Gly content was significantly higher in Chanee than Monthong (*p* < 0.01) [8]. Therefore, *DzLAP1* is not cultivar-dependent and does not account for the relative differences between Chanee and Monthong in terms of fruit odour intensity. The competitive ethylene inhibitor 1-MCP significantly repressed *DzLAP1* during postharvest ripening (*p* < 0.05) (Fig. 3b). Thus, *DzLAP1* might play an important role in this process and *LAP1* may be associated with the early stages of ripening. In contrast, *LAP-N*s in other plant species are constitutively expressed in all organs [34, 43]. We compared *LAP* expression during tomato fruit ripening and found that the *LAP-A* levels were slightly lower at the breaker or ethylene-producing stages than they were in mature green fruit (Supplementary Fig. S2a). Tomato *LAP-N* is constitutively expressed at all fruit developmental stages (Supplementary Fig. S2b). Therefore, *LAP*s are not implicated in tomato fruit development or ripening.

Cys-Gly dipeptidase activity was observed in crude durian fruit extract (Supplementary Fig. S3) and in co-infiltrated N. benthamiana leaves overexpressing DzLAP1 (Fig. 4). Cys-Gly hydrolytic activity was higher in midripe than unripe durian pulp extracts (Supplementary Fig. S3). This finding was consistent with DzLAP1 upregulation at the midripe stage (Fig. 3a). Thus, Cys-Gly dipeptidase was functional in planta. The observed Cys-Gly dipeptidase activity of DzLAP1 in planta upholds our aforementioned hypothesis and suggests that the Cys generated by Cys-Gly hydrolysis is converted into sulphur volatiles contributing to the malodour of ripening durian fruit. Hence, the γ-glutamyl cycle is essential for recycling GSH and its amino acid constituents and for durian fruit ripening. Nevertheless, an alternative pathway to GSH recycling has been suggested in other plants. In ripening tomato fruit, the oxidised glutathione (GSSG)-catabolising enzyme γ-glutamyl transpeptidase (GGT) plays a major role in glutathione degradation that releases Cys-Gly and Glu [44]. In Arabidopsis, an increased GSH content and a drop in Cys-Gly content were observed in the ggt1 knockout line when compared with those of wild type plants [45]. Unlike tomato and Arabidopsis, however, durian fruit is rich in sulphur. For this reason, γ-glutamyl cycle in durian might have evolved to be more active during ripening.

An in vitro biochemical assay of rDzLAP1 (Fig. 5) disclosed that LAPs have pH optima in the range of 8.0–11.0 and metal ion dependency [29, 46, 47]. DzLAP1 has high catalytic efficiency (*k*_*cat*_/K_m_) for Cys-Gly (K_m_ = 1.6 mM). Therefore, DzLAP1 is a metalloenzyme and a Cys-Gly dipeptidase. The aforementioned K_m_ resembles those for other Cys-Gly peptidases such as AtLAP1 [15], yeast Dug1p [17], and bacterial TdLAP [14] all of which were ≤ 1.3 mM. Thus, the affinity of LAP for Cys-Gly corresponds to the millimolar physiological GSH concentration range in living cells [48, 49]. Durian is more abundant in GSH than many other fruits and vegetables [8, 50]. For this reason, the cellular GSH level in durian should be higher than the general physiological concentration of this substance and Cys-Gly dipeptidases have highly conserved activity across species. As for other degradable α-linked dipeptides, DzLAP1 could not hydrolyse γ-Glu-Cys-Gly (Table 1). Consequently, this enzyme might have specificity for α-peptide bonds. However, earlier studies did not investigate the affinities of LAPs for γ-linked dipeptides.

Subcellular protein localisation can elucidate the correlations between the native functions and the physiological substrates of an enzyme. DzLAP1 is a dual-target protein in the chloroplasts and the cytosol (Fig. 6). However, it harbours a plastidial transit peptide sequence. *DzLAP1* transcripts may have an alternative codon to initiate ribosomal translation that bypasses the first start codon [34] and/or forms secondary RNA structures in the sequences adjacent to it [51]. Delta-2-isopentenyl pyrophosphate:tRNA isopentenyl transferase (MOD5) is encoded by a single gene, has two translational initiation sites, and is localised to the mitochondria, cytosol, and nucleus [52]. Plastidial DzLAP1 may recycle protein and perform other tasks. As chloroplasts participate in cellular metabolism, they may respond to various stressors [53]. The present study did not clarify the functions of plastidial DzLAP1. However, we propose that, like AtLAPs, it is a molecular chaperone preventing misfolded protein accumulation and other adverse effects [36]. Partial DzLAP1 localisation to the chloroplasts enhances its chaperone and/or protease activity in specific suborganellar compartments. Similar observations were reported for CRP-like protein (Clp) and filamentous temperature-sensitive protein (FtsH). These are major conserved ATP-dependent chaperone and degradative proteases in the stroma and on the thylakoid membranes, respectively [54]. Further experimentation is needed to confirm the role of DzLAP1 in the plastids during fruit ripening.

As Cys-Gly has pro-oxidant activity, its concentration must be regulated. Cys-Gly promotes the formation of reactive oxygen species (ROS) such as hydrogen peroxide and superoxide anion in the presence of certain metal ions [55]. The next step is the oxidation of highly reduced thiols such as GSH [56]. Cytosolic localisation and the enzyme kinetics of DzLAP1 suggest that it hydrolyses Cys-Gly in the cytoplasmic γ-glutamyl cycle and controls cytosolic Cys-Gly levels. Cys-Gly may also be a substrate for cytosolic DzLAP1 in vivo. In this manner, DzLAP1 regulates Cys-Gly concentration. As *DzLAP1* is localised to the cytosol and expressed where ethylene and sulphur volatiles accumulate, it might participate in durian fruit ripening. In fact, the slightly alkaline pH of the cytoplasm [57] and the chloroplast stroma [58, 59] is conducive to DzLAP1 activity.

**Fig. 7:**
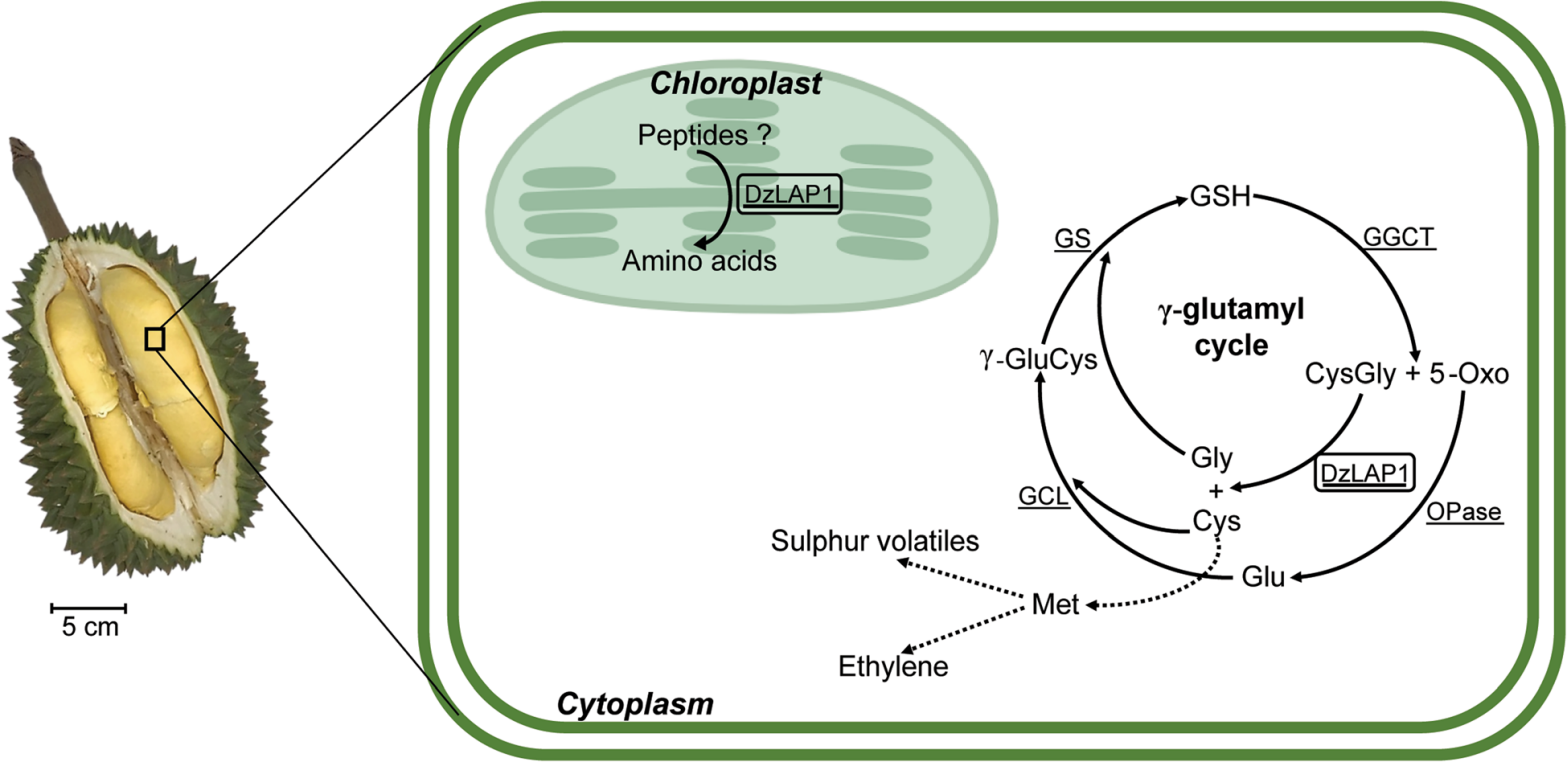
Schematic representation of putative DzLAP1 functions in durian fruit pulp cytosol and chloroplast. DzLAP1 resides in both the chloroplast and the cytoplasm in the γ-glutamyl cycle (boxes). Broken lines indicate that at least one reaction is involved. All enzymes are underlined. DzLAP1: leucylaminopeptidase1; GGCT: γ-glutamylcyclotransferase; OPase: oxoprolinase; GCL: glutamate cysteine ligase; GS: GSH synthase; 5-Oxo: 5-oxoproline

DzLAP1 and the LAP-N of *Arabidopsis* and Solanaceae all have similar catalytic, substrate, and metal ion-binding residues. However, *DzLAP1* expression differs from those of the latter genes. Durian accumulates high levels of sulphur volatiles that impart a strong flavour and unique odour to the fruit. However, the levels of these thiols must be tightly controlled. Expression of the genes encoding sulphur-metabolising enzymes must be regulated during fruit ripening. The *DzLAP1* promoter region may have been modified to produce the optimal DzLAP1 content, limit the amount of Cys-Gly, catalyse Cys recycling, and generate sulphur volatiles and ethylene during fruit ripening. A similar finding was reported for durian-specific upregulation of the methionine γ-lyase gene [5]. The latter is responsible for sulphur volatile production in plants and bacteria [60, 61]. An isoform of this enzyme associated with fruit ripening has been identified [5]. Figure 7 is a schematic diagram summarising the putative functions of DzLAP1 in durian fruit pulp cytoplasm and chloroplasts.

## Conclusions

Based on durian cultivar Musang King genomic data, we identified and characterised the LAP-N gene encoding leucylaminopeptidase (LAP). This enzyme is highly expressed in durian fruit pulp and was designated *DzLAP1*. We established that *DzLAP1* is active in the early stages of durian fruit ripening. However, it is not cultivar-dependent and may not be responsible for the fact that ripe Chanee durian fruit has a stronger odour than ripe Monthong durian fruit. An *in planta* assay in *Nicotiana benthamiana* leaves demonstrated Cys-Gly dipeptidase activity. The enzyme kinetics and subcellular localisation of *DzLAP1* indicate that it has in vivo Cys-Gly dipeptidase activity*.* Its presence in the cytosol suggests that it participates in the γ-glutamyl cycle and is adjacent to intracellular ethylene and sulphur volatile production sites. The plastidial DzLAP1 isoform may participate in protein turnover and/or protection. The present study partially elucidated the mechanisms of sulphur metabolism in plant tissues accumulating high levels of sulphur volatiles.

## Methods

### Plant materials and growth conditions

Mature durian fruit were obtained from two different commercial orchards in Thailand. Chanee fruit were harvested at 95 d after anthesis from a commercial orchard (12°09′56.6″N 102°41′13.1″E) in Trat Province in early April 2017. The samples were maintained at room temperature (30 °C) before being peeled on days 1, 2, and 4 (unripe, midripe, and ripe postharvest stages, respectively). Monthong fruit were harvested at day 105 after anthesis from a commercial orchard (12°40′39.2″N 102°05′35.2″E) in Chanthaburi Province. Monthong served as a representative sample for gene expression analysis. Its three different ripening stages were also evaluated but their timings (days) slightly differed from those of Chanee. Unripe, midripe, and ripe Monthong fruit samples were peeled and analysed at 1 d, 3 d, and 5 d, respectively, after storage at room temperature [6]. No special permission was necessary to collect such samples. The collection of plant materials complies with national and international guidelines.

To investigate the association between *DzLAP1* and durian fruit ripening, unripe Monthong samples were treated either with ethephon or 1-methylcyclopropene (1-MCP). These synthetic phytohormones have opposite modes of action. Ethephon is converted to ethylene which enhances ripening. In contrast, 1-MCP is an ethylene antagonist and delays ripening. The treated samples were compared with controls naturally ripened according to the method of Khaksar et al. [6]. Three biological replicates were used and each comprised a single durian fruit harvested from a separate tree.

Crude extract from durian pulp was used to determine Cys-Gly dipeptidase activity. Durian cultivar Chanee was obtained from a local market in Nonthaburi Province, Thailand. Postharvest samples were collected at the unripe and midripe stages. Three biological replicates were used and each consisted of a single lobe harvested from a separate durian fruit.

*Nicotiana benthamiana* plants were raised for agroinfiltration. Seeds were sown on peat moss and the seedlings were grown at 25 °C under a 16 h/8 h light/dark photoperiod and 4500 lx (artificial light). Two-week-old plants were individually transplanted to pots and raised under the same conditions for another 2 wks.

### Phylogenetic analysis and putative LAP identification in durian fruit

The protein sequences of LAPs harbouring Cys-Gly dipeptidase activity in *Treponema denticola* (accession no. WP_010698434.1) [14] and *Arabidopsis thaliana* (AtLAP1 and AtLAP3; accession nos. P30184.1 and Q8RX72.1, respectively) [15] served as queries for a BLAST search against the *D. zibethinus* cultivar Musang King NCBI database. The MaGenDB database [62] confirmed *DzLAP* isoforms.

To establish the phylogenetic relationships among LAPs, the amino acid sequences of the putative DzLAPs and other LAPs deposited in NCBI were subjected to ClustalW multiple alignment. A neighbour joining (NJ) tree was created with MEGA v. 7 [63] using 1000 bootstrap replicates.

### Determination of tissue-specific *DzLAP1* expression

A search of the *DzLAP*s against the genomic data for durian cultivar Musang King disclosed two candidate genes (accession nos. XM_022894525.1 (LOC111299369) and XM_022874012.1 (LOC111284913)) annotated as *DzLAP1-like* and named *DzLAP1_MK* and *DzLAP2_MK*, respectively. Attention was directed to the durian fruit pulp as it accumulated several sulphur volatiles. *DzLAP1* expression was analysed in silico in various fruit tissues. To compare relative *DzLAP* expression in different tissues, normalised total read counts (RCs) derived from Illumina reads were obtained from the Sequence Read Archive (SRA) resource and processed according to the method of Khaksar et al. [6]. RNA-seq data were obtained for SRX3188225 (root), SRX3188222 (stem), SRX3188226 (leaf), and SRX3188223 (aril/pulp) [5]. A heatmap based on the RCs was constructed using MetaboAnalyst v. 4.0 [64].

### Gene expression analysis by qRT-PCR

Total RNA was isolated from Chanee and Monthong durian cultivar pulps with PureLink® plant RNA reagent (Thermo Fisher Scientific, Waltham, MA, USA) following the manufacturer’s instructions. DNase-treated RNA sample quantity and integrity were assessed. Approximately 1 μg total RNA was reverse-transcribed to cDNA with a RevertAid first-strand cDNA synthesis kit (Thermo Fisher Scientific, Waltham, MA, USA). Gene-specific primers listed in Supplementary Table S1. The qRT-PCR elucidated *DzLAP1* expression in unripe, midripe, and ripe durian fruit. The reactions were performed in 10 μL total volume in a 96-well PCR plate. The cDNA and primers were combined with Luna® universal qPCR master mix (New England Biolabs, Ipswich, MA, USA). PCR was run in a CFX Connect™ real-time PCR detection system coupled to CFX Manager™ (Bio-Rad Laboratories, Hercules, CA, USA). Single amplicon production was verified by melting curve analysis. Relative gene expression levels were calculated by the 2^−ΔΔCt^ method [65] based on the cycle threshold (Ct) of the gene relative to the reference gene elongation factor 1 alpha (*EF-1α*). There were three independent biological replicates in the qRT-PCR. Gene expression analyses were also conducted on the ethephon- and 1-MCP-treated samples and the naturally un/ripened samples (controls).

### *In planta* Cys-Gly dipeptidase activity assay

To determine Cys-Gly dipeptidase activity in durian fruit pulp, crude enzyme was extracted from it at the unripe and midripe stages. Pulp samples were separately collected, frozen in liquid nitrogen, and pulverised in the MM400 mixer mill (Retsch GmbH, Haan, Germany) at 30 Hz for 1 min. Then 250 mg of each sample was dissolved in 2.5 mL lysis buffer (50 mM K_3_PO_4_, pH 8.0) and gently mixed at 4 °C for 1 h. The samples were centrifuged at 14,000×*g* and 4 °C for 5 min and the supernatant was collected. An enzyme activity assay was performed by incubating durian fruit pulp extract with 20 mM Cys-Gly in reaction buffer (50 mM K_3_PO_4_ buffer (pH 8.0) + 1 mM MgCl_2_) at 30 min, 1 h, and 3 h. The enzyme activity was measured by a modified acidic ninhydrin method [39]. Briefly, 50 μL of each enzyme reaction system was terminated with 50 μL glacial acetic acid followed by 50 μL acidic ninhydrin solution (250 mg ninhydrin in 6 mL glacial acetic acid + 4 mL HCl), boiled for 9 min, and cooled with tap water. The pink endpoint indicated the reaction between the released Cys and ninhydrin under acidic conditions. Colour intensity was measured spectrophotometrically at A_560_ (BioTex, Winooski, VT, USA). Three independent biological replicates were used.

*In planta* Cys-Gly dipeptidase activity was also investigated. Full-length *DzLAP1* was amplified with Phusion Hot Start II high-fidelity DNA polymerase (Thermo Fisher Scientific, Waltham, MA, USA) using Chanee cDNA as a template. The gene-specific primers (excluding the stop codon) listed in Supplementary Table S1 were used in the PCR. The PCR product was cloned into a pCR™8/GW/TOPO® TA cloning vector (Invitrogen, Carlsbad, CA, USA) and generated pTOPO-*DzLAP1*. The latter was then sequenced. *DzLAP1* was transferred to the pEAQ3 destination vector and fused at the *C*-terminal with 6× His [66] via Gateway® LR Clonase® II (Invitrogen, Carlsbad, CA, USA). The pEAQ-*DzLAP1* product was transformed into *Agrobacterium tumefaciens* GV3101 by electroporation.

*A. tumefaciens* bearing the *DzLAP1-6xHis* or the empty pEAQ vector (control) was infiltrated into 4 wk. plants. Briefly, cells from each culture were washed and suspended in MM buffer (10 mM MES buffer + 10 mM MgCl_2_; pH 5.6). The cell suspension was adjusted at A_600_ to OD = 0.6. Acetosyringone was added to make up 200 μM final concentration and the suspension was kept in the dark at 30 °C for 2 h before infiltration. At day 3 after agroinfiltration, the leaves were infiltrated with 15 mM Cys-Gly, incubated for 1 h [67], freeze-dried, and kept in a dry place at 30 °C until subsequent metabolite analysis by HPLC. To measure the foliar Cys-Gly levels, the co-infiltrated leaves were pulverised in the MM400 mixer mill (Retsch GmbH, Haan, Germany) at 30 Hz for 1 min. Each sample was suspended in 0.1 M HCl extraction buffer at 1 mg/50 μL. Then, 10 mM *N*-acetylcysteine (internal standard) was added and mixed by shaking at 250 rpm and 37 °C for 2.5 h. The samples were centrifuged at 14,000×*g* and 30 °C for 5 min. The soluble fraction was transferred to a new microcentrifuge tube, combined with acetonitrile (1:1), and centrifuged at 14,000×*g* and 30 °C for 5 min. The pellet was removed and the supernatant was dried with a CentriVap benchtop vacuum concentrator (Labconco Corp., Kansas City, MO, USA). The dried samples were re-dissolved in 200 μL deionised H_2_O, passed through a 0.22-μm syringe filter, and subjected to HPLC analysis. Ten microlitres of each sample was injected into a C18 column (250 mm × 4.6 mm; Phenomenex, Torrance, CA, USA) using acetonitrile (5%) in 2% perchloric acid as a mobile phase. The flow rate was 1 mL/min and the temperature was 40 °C. The Cys-Gly peak was detected at 210 nm [15]. Three independent biological replicates were used and each consisted of a separate leaf.

### *DzLAP1* cloning and expression in *Escherichia coli*

The putative *DzLAP1* was amplified with Phusion Hot Start II DNA polymerase (Thermo Fisher Scientific, Waltham, MA, USA) and midripe Chanee durian cultivar cDNA served as a template. The PCR temperature profile was as follows: initial denaturation at 98 °C for 30 s; 30 cycles of 98 °C for 10 s; 57 °C for 10 s; 72 °C for 1 min; and a final extension at 72 °C for 5 min. Gene-specific forward and reverse primers were designed according to the *DzLAP1_MK* sequence (Supplementary Table S1). The signal sequences predicted by the ChloroP1.1 and TargetP servers were excluded. The putative *DzLAP1* PCR product was excised with restriction enzymes (FastDigest™; Thermo Fisher Scientific, Waltham, MA, USA) and cloned into a pET21b vector (Merck KGaA, Darmstadt, Germany). The product was pET21b-*DzLAP1* in-frame with 18 nucleotides encoding 6× His residues at the *C*-terminus. It was transformed into *E. coli* TOP10 (K2500–20; Invitrogen, Carlsbad, CA, USA). Bacterial colonies were raised on LB agar supplemented with 1 mg mL^− 1^ ampicillin and analysed by colony PCR. The nucleotide sequences of the positive clones were verified by 1st BASE DNA sequencing.

### Recombinant DzLAP1 (rDzLAP1) production and purification

The pET21b-*DzLAP1* was transformed into the T7 host *E. coli* SoluBL21 (DE3). Cells harbouring the recombinant plasmid were incubated overnight in LB broth supplemented with 1 mg mL^− 1^ ampicillin. The starter culture was inoculated into fresh LB broth supplemented with 1 mg mL^− 1^ ampicillin and incubated at 37 °C with shaking at 250 rpm until OD_600_ = 0.4–0.5. The rDzLAP1 was generated by induction with 1 mM isopropyl β-*D*-1-thiogalactopyranoside (IPTG) at 30 °C and shaking at 250 rpm for 17 h. The cells were harvested by centrifugation at 5000×*g* and 37 °C for 10 min, suspended in buffer A (25 mM K_3_PO_4_ buffer + 0.3 M NaCl; pH 7.2), and lysed by ultrasonication. Soluble intracellular proteins were collected by centrifugation at 7500×*g* and 4 °C for 30 min, analysed by western blot (6× His epitope tag antibody; Thermo Fisher Scientific, Waltham, MA, USA), and stored at 4 °C until purification.

The crude extract was loaded onto a HisTrap™ column (Merck, Darmstadt, GER) pre-equilibrated with buffer A. The column was washed with excess buffer A and the rDzLAP1 was eluted with buffer B (25 mM K_3_PO_4_ buffer + 0.3 M NaCl + 150 mM imidazole; pH 7.2). The samples were analysed by SDS-PAGE and western blot. The pooled purified rDzLAP1 fraction was dialysed against 25 mM K_3_PO_4_ buffer (pH 7.2). The protein concentration was determined by a modified Bradford assay [68]. The reference protein standard was BSA.

### Enzymatic rDzLAP1 assay

The metal ion dependency of rDzLAP1 was evaluated. The enzyme was incubated at 37 °C for 15 min with 7.5 mM Cys-Gly in 25 mM K_3_PO_4_ buffer (pH 7.2) in the presence of 0 mM or 1 mM Ca^2+^, Zn^2+^, Mg^2+^, Ni^2+^, or Mn^2+^. The total reaction volume was 50 μL. Enzyme activity was measured by a modified acidic ninhydrin method [39]. To determine the optimum enzyme pH, 50-μL reaction systems were prepared by incubating rDzLAP1 with 7.5 mM Cys-Gly and 1 mM Mg^2+^ at various pH (acetate buffer, pH 4–6; phosphate buffer, pH 6–8; Tris-HCl buffer, pH 8–9.5; glycine-NaOH buffer, pH 9.5–11) at 37 °C for 15 min. Maximum enzyme activity was defined as 100% relative activity.

To assess enzyme kinetics, 3.5 μg purified rDzLAP1 was incubated with 0–10 mM Cys-Gly, γ-Glu-Cys, GSH, Met-Gly, or Leu-Gly in the presence of 1 mM MgCl_2_ in 25 mM K_3_PO_4_ buffer (pH 8.0) at 37 °C for a specific length of time. The reactions were terminated with 0.13 N HCl. For γ-Glu-Cys and GSH, the reactions were evaluated by a modified acidic ninhydrin assay. For Cys-Gly, Met-Gly, and Leu-Gly, the reactions were analysed by monitoring the decrease in absorbance of the free peptides at A_230_, with minor modifications [69]. Enzyme kinetics were measured with OriginPro® 2017 (OriginLab Corp., Northampton, MA, USA).

### Subcellular localisation

Full-length *DzLAP1* was amplified and cloned into a pCR™8/GW/TOPO® TA vector (Invitrogen, Carlsbad, CA, USA) according to the *in planta* Cys-Gly dipeptidase activity assay. The pTOPO-*DzLAP1* product was then sequenced. The *DzLAP1* was transferred to the pGWB5 destination vector and fused at the *C*-terminal with green fluorescent protein (GFP) [70] via Gateway® LR Clonase® II (Invitrogen, Carlsbad, CA, USA). The pGWB5-*DzLAP1* product was transformed into *A. tumefaciens* GV3101 by electroporation.

*Agrobacterium tumefaciens* bearing the *DzLAP1-GFP* construct and *A. tumefaciens* with the silencing suppressor *p19* gene [71] were co-infiltrated into 4-wk plants as previously described, with some modifications. Briefly, cells from each culture were washed and suspended in MM buffer (10 mM MES buffer + 10 mM MgCl_2_; pH 5.6). Cell suspensions harbouring *DzLAP1* and *p19* were adjusted at A_600_ to OD = 0.8 and 0.6, respectively, and combined in a 1:1 ratio. Acetosyringone was added to the mixture to a final concentration of 200 μM and the suspension was maintained in the dark at room temperature for 2 h before infiltration. At day 3 after infiltration, autofluorescence was visualised under a FluoView® FV10i-DOC confocal laser scanning microscope (Olympus Corp., Tokyo, Japan). GFP, chloroplast autofluorescence, and phase-contrast detection excitation/emission were recorded at 473/510 nm, 559/600 nm, and 559/600 nm, respectively.

### Statistical analysis

All data were analysed with SPSS Statistics® v. 22.0 (IBM Corp., Armonk, NY, USA). One-way ANOVA identified significant differences among mean enzyme activity levels in the absence and presence of metal ions. Cys-Gly dipeptidase gene expression levels and enzyme activity at various ripening stages and in response to different ripening regulators were analysed by Tukey’s HSD multiple comparisons test (*p* < 0.05). Student’s *t*-test (*p* < 0.05) identified significant differences between *N. benthamiana* leaves overexpressing *DzLAP1* and the control in terms of *in planta* Cys-Gly dipeptidase activity*.*

## Supplementary Information

**Additional file 1: Supplementary Table S1.** Primers used in the present study. Restriction sites are underlined.

**Additional file 2: Supplementary Fig. S1.** Tissue-specific *DzLAP* expression profiles in Musang King durian fruit pulp. *DzLAP1_MK* and *DzLAP2_MK* expression levels in fruit pulp, stem, leaf, and root were analysed by RNA-seq. Red: higher gene expression level; blue: lower gene expression level. Data were sum-normalised, log-transformed, and autoscaled.

**Additional file 3: Supplementary Fig. S2.** Relative *LAP-A* (a) and *LAP-N* (b) expression at various developmental stages of tomato fruit. *LAP-A* (Solyc12g010020) (a) and *LAP-N* (Solyc12g010040) (b) expression levels in tomato (*Solanum lycopersicum*) were determined from Illumina-based and RPKM-normalised data [72] and represented in Tomato eFP Browser v. 2.0. Mature green, breaker, and breaker + 10 d tomato fruit ripening stages were compared.

**Additional file 4: Supplementary Fig. S3.** Determination of Cys-Gly dipeptidase activity in durian pulp extract. Twenty-microlitre unripe (black bars) and midripe (white bars) durian fruit extracts were incubated with 20 mM Cys-Gly in 50 mM K_3_PO_4_ buffer (pH 8.0) in the presence of 1 mM Mg^2+^ at 37 °C for 30 min, 1 h, and 3 h. The total reaction volume was 50 μL. Enzyme activity was measured spectrophotometrically by a modified acidic ninhydrin method at 560 nm (A_560_). Bars: means ± standard deviation (SD) of three independent biological replicates. Different letters indicate significant differences according to Tukey’s HSD multiple-range test (*p* < 0.05).

## Data Availability

The datasets used and/or analysed during the current study are available from the corresponding author on reasonable request.

## Abbreviations

GSH: Glutathione
γ-EC: γ-glutamylcysteine
Cys-Gly: Cysteinylglycine
Cys: Cysteine
LAP: Leucylaminopeptidase
*DzLAP*: *Durio zibethinus* L. leucylaminopeptidase gene
