# Supplementary material for: Fruit ripening-associated leucylaminopeptidase with cysteinylglycine dipeptidase activity from durian suggests its involvement in glutathione recycling
Source: BMC Plant Biol. 2021 Feb 1;21:69. doi: 10.1186/s12870-021-02845-6 (PMC7852106; doi:10.1186/s12870-021-02845-6)
Supplement: Supplementary file 1 — Additional file 1: Supplementary Table S1. Primers used in the present study. Restriction sites are underlined. [file 12870_2021_2845_MOESM1_ESM.docx]

**Supplementary Table S1** Primers used in the present study. Restriction sites are underlined

| **Primer name** | **Sequence (5**′ **→ 3**′**)** |
| --- | --- |
| **Gene expression analysis** |  |
| Frt1_DzLAP | CCACTGGAGGAAAGCTATTG |
| Rrt1_DzLAP | GAACAGAGCTGCAGTGATAG |
| **Subcellular localisation** |  |
| F_DzLAP | ATGGTCGCCACCATTGTAGCGTCCTG |
| R_DzLAP | AGAAGAGTTTTTCAGAACCCATTCC |
| **Recombinant expression** |  |
| F_matureDzLAPNdeI | GGAATTCCATATGTGTTCTCGAAGAGCCAAGTTCATGG |
| R_DzLAPXhoI | CCGCTCGAGAGAAGAGTTTTTCAGAACCCATTCC |
