# Supplementary figures and images for: Fruit ripening-associated leucylaminopeptidase with cysteinylglycine dipeptidase activity from durian suggests its involvement in glutathione recycling
Source: BMC Plant Biol. 2021 Feb 1;21:69. doi: 10.1186/s12870-021-02845-6 (PMC7852106; doi:10.1186/s12870-021-02845-6)

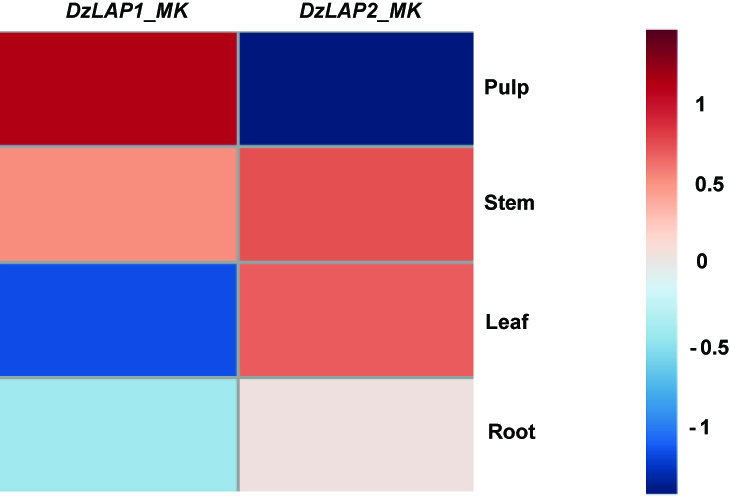

Supplement: Supplementary file 2 — Additional file 2: Supplementary Fig. S1. Tissue-specific DzLAP expression profiles in Musang King durian fruit pulp. DzLAP1_MK and DzLAP2_MK expression levels in fruit pulp, stem, leaf, and root were analysed by RNA-seq. Red: higher gene expression level; blue: lower gene expression level. Data were sum-normalised, log-transformed, and autoscaled. [file 12870_2021_2845_MOESM2_ESM.tif]

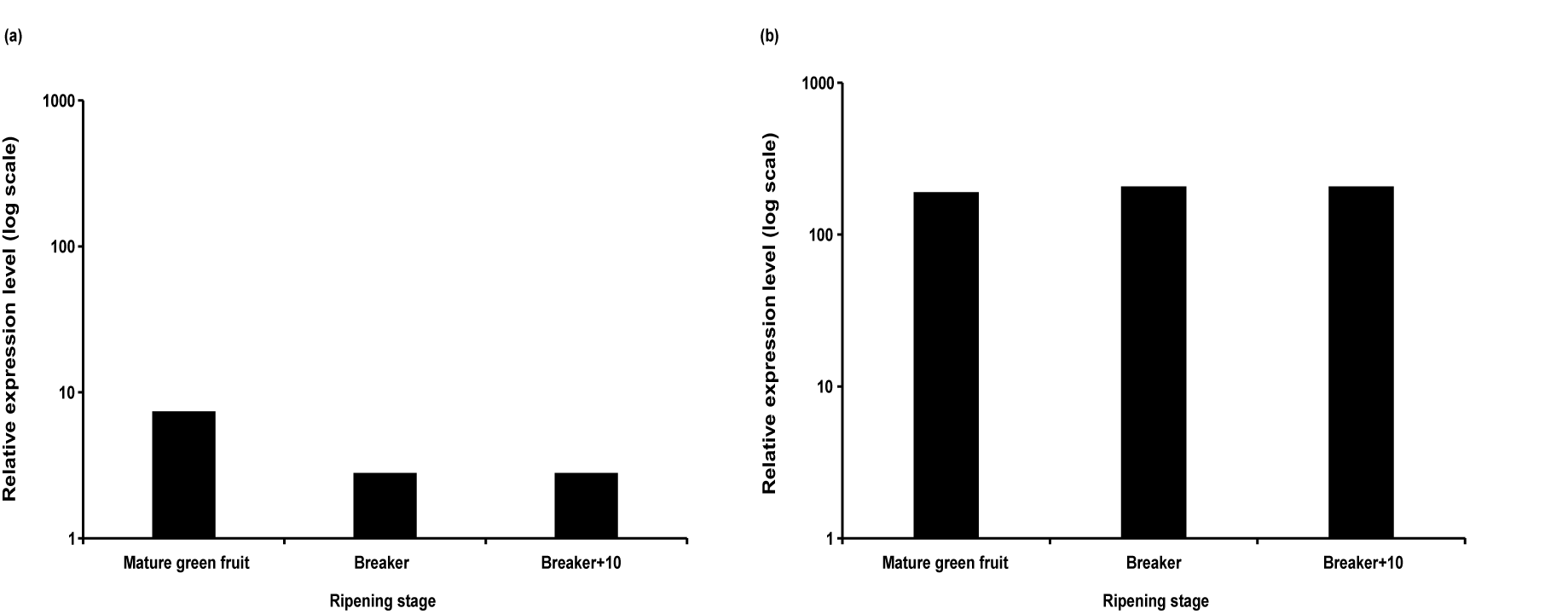

Supplement: Supplementary file 3 — Additional file 3: Supplementary Fig. S2. Relative LAP-A (a) and LAP-N (b) expression at various developmental stages of tomato fruit. LAP-A (Solyc12g010020) (a) and LAP-N (Solyc12g010040) (b) expression levels in tomato (Solanum lycopersicum) were determined from Illumina-based and RPKM-normalised data [72] and represented in Tomato eFP Browser v. 2.0. Mature green, breaker, and breaker + 10 d tomato fruit ripening stages were compared. [file 12870_2021_2845_MOESM3_ESM.tif]

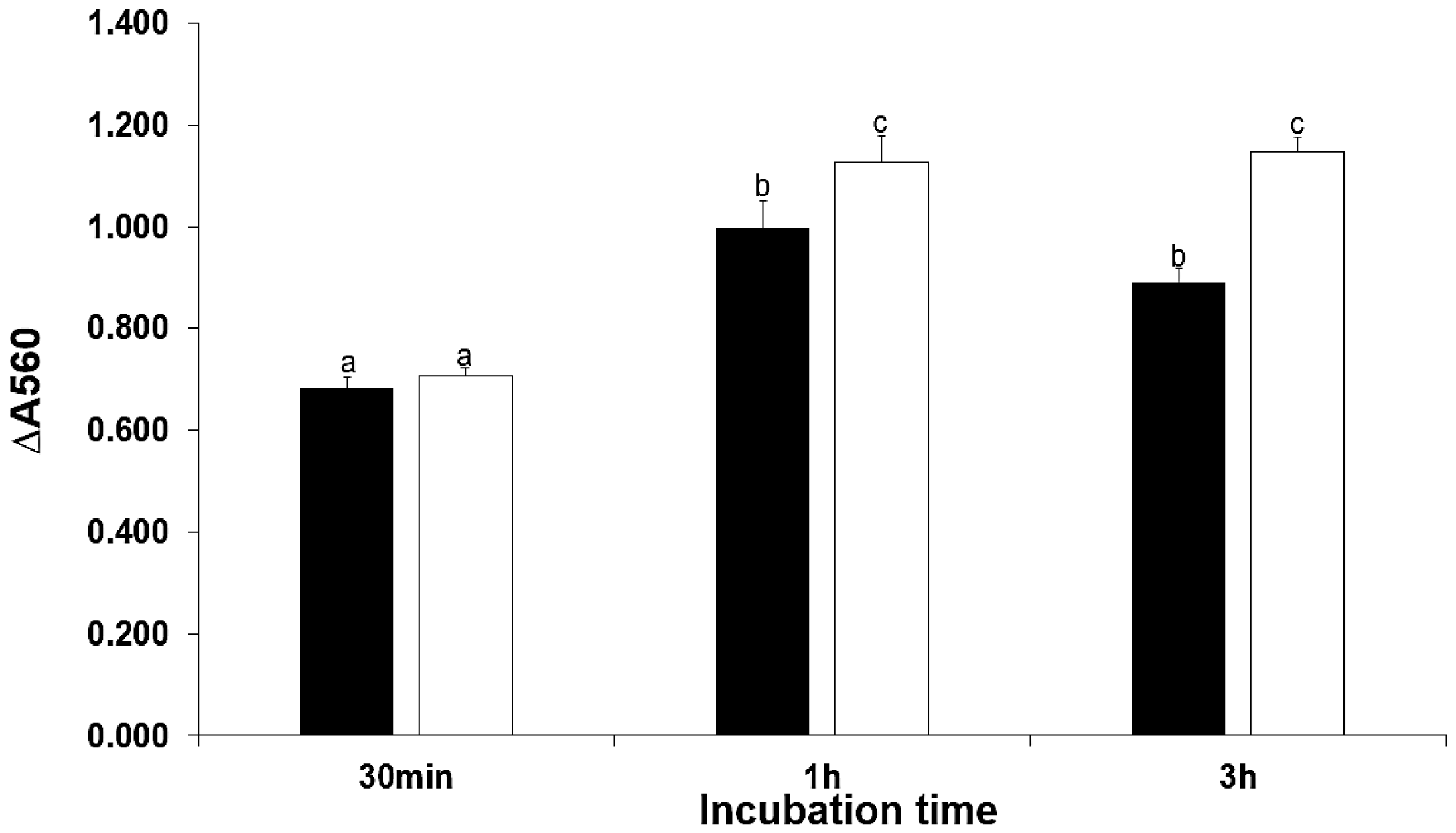

Supplement: Supplementary file 4 — Additional file 4: Supplementary Fig. S3. Determination of Cys-Gly dipeptidase activity in durian pulp extract. Twenty-microlitre unripe (black bars) and midripe (white bars) durian fruit extracts were incubated with 20 mM Cys-Gly in 50 mM K3PO4 buffer (pH 8.0) in the presence of 1 mM Mg2+ at 37 °C for 30 min, 1 h, and 3 h. The total reaction volume was 50 μL. Enzyme activity was measured spectrophotometrically by a modified acidic ninhydrin method at 560 nm (A560). Bars: means ± standard deviation (SD) of three independent biological replicates. Different letters indicate significant differences according to Tukey’s HSD multiple-range test (p < 0.05). [file 12870_2021_2845_MOESM4_ESM.tif]
